# Nanoparticle-Reinforced Hydrogel with a Well-Defined Pore Structure for Sustainable Drug Release and Effective Wound Healing

**DOI:** 10.1021/acsabm.4c01659

**Published:** 2025-02-07

**Authors:** Ziyi Zhang, Siyu Yang, Feixue Mi, Yicheng Yang, Qi Song, Yibo Gao, Changfeng Wu, Weijia Wen

**Affiliations:** † Division of Emerging Interdisciplinary Areas, The Hong Kong University of Science and Technology, Clear Water Bay, Kowloon, Hong Kong 000000, China; ‡ Thrust of Advanced Materials, The Hong Kong University of Science and Technology (Guangzhou), Nansha, Guangzhou 511400, China; § Department of Physics, The Hong Kong University of Science and Technology, Clear Water Bay, Kowloon, Hong Kong 000000, China; ∥ Department of Biomedical Engineering, Southern University of Science and Technology, Shenzhen 518055, China; ⊥ Shenzhen Shineway Technology Corporation, Shenzhen 518048, China

**Keywords:** wound healing, composite hydrogels, nanoparticle drugs, anti-inflammation, synergy therapy

## Abstract

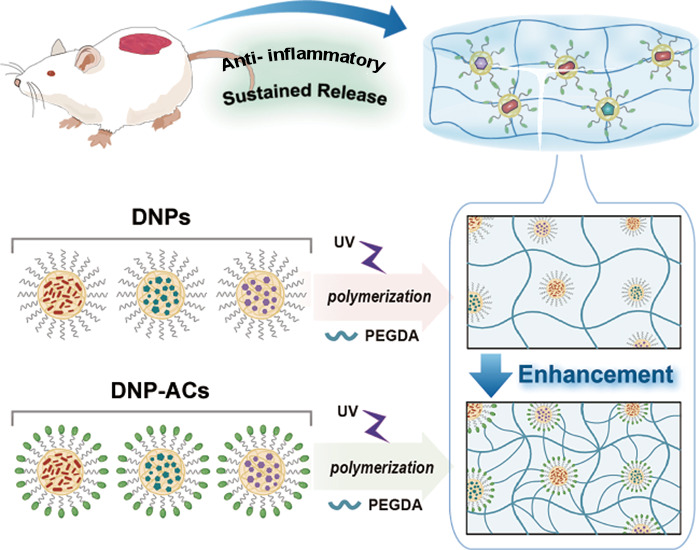

Impaired chronic wounds are a common complication of diabetes. Inhibited angiogenesis and dysfunctional inflammation render diabetic wound healing a critical challenge. Herein, a sustainable therapeutic composite hydrogel is presented for diabetic wound healing, consisting of a cocktail formulation of anti-inflammatory and local anesthetic nanoparticles incorporated into a composite hydrogel. The surface-modified drug nanoparticles are loaded into the biocompatible hydrogels and cross-linked with a gel precursor to enhance the structure. The sustainable delivery system achieves more than 90% drug release, with a total therapy duration tunable from 4 to 72 h. Through the long-lasting anti-inflammatory and analgesic effects of the composite hydrogel, diabetic wounds are swiftly transitioned into the proliferation phase, augmenting the survival and migration of keratinocytes and facilitating neovascularization and collagen alignment in diabetic wounds. These effects significantly improve the wound healing rate and skin regeneration process, achieving a healing rate that is 17 times that of untreated wounds. This study demonstrates that the hydrogel platform loaded with cocktail drug nanoparticles is promising for the rapid healing of diabetic wounds.

## 1 Introduction

Diabetes is a common metabolic disease that afflicts 6.1% of the global population.^1^ Chronic inflammation is a frequent hazard for diabetic patients and significantly hinders the wound recovery process.^2^ Prolonged local infection and inflammation impede angiogenesis, epithelialization, and anti-infective activity of neutrophils. Chronic wounds make the foot susceptible to ulcers, gangrene, and even amputation, imposing substantial psychological and economic burdens on patients.^3,4^ However, it remains challenging for traditional wound dressings to meet the complex healing process. Currently, standard clinical wound dressings (e.g., gauze, film) have insufficient adaptability to the diabetic wound environment, as they neither continuously release active substances nor accelerate the wound healing process, often encountering the risk of adherence and damage to diabetic wounds.^5−8^ Hydrogels, which contain large quantities of water, provide a moist environment for patient comfort, holding promise as wound dressings.^5,9−14^ Hydrogels have structural similarities to soft tissue and can incorporate therapeutic ingredients that promote diabetic wound healing.^15−23^ Researchers have recently developed advanced platforms for effective wound healing through the use of injectable composite hydrogels that incorporate nanospheres and stem cell-derived exosomes.^24−26^ These innovative materials enhance the healing process by promoting cell proliferation and tissue regeneration. Composite hydrogels with enhanced functionalities have been demonstrated by different approaches such as direct mixing with nanoparticle suspension,^27,28^ connecting the drug to the linkage via addition reaction,^29−31^ and incorporating hydrophobic domains to bind the drug.^32^ However, these hydrogel platforms have limitations, including uncontrolled release, complex preprocessing reaction conditions, and altered hydrophilicity of the hydrogel^33−36^

**Scheme 1:**
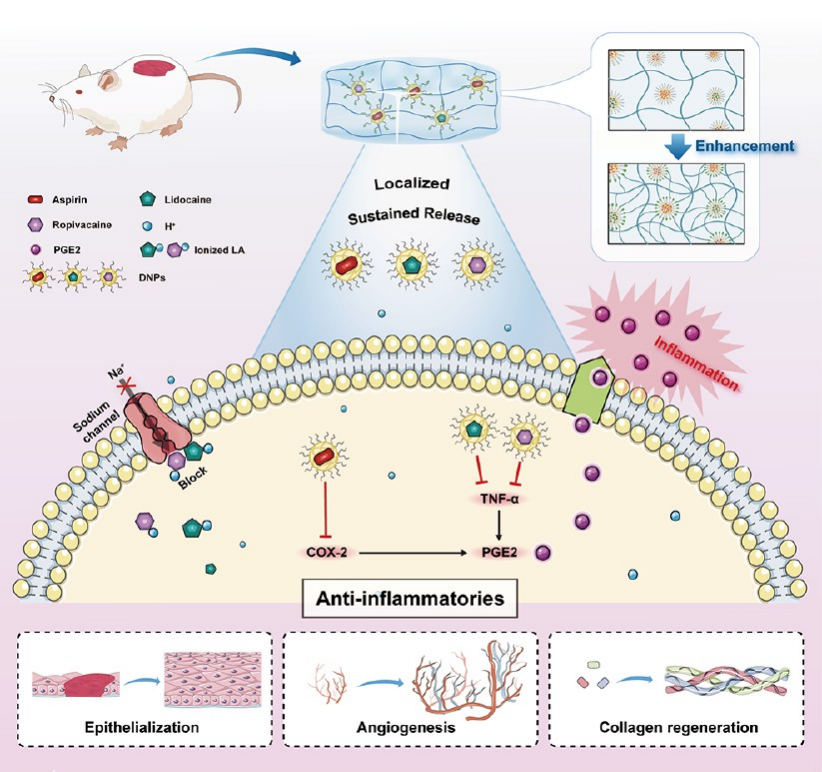
Illustration of the Anti-inflammatory Mechanism of a Sustainable Therapeutic Platform The local hydrogel releases a cocktail combination of anti-inflammatory drugs and LAs on the wounds of diabetic mice. The structure and sustained release of the hydrogel are enhanced by the surface properties of the drug nanoparticles during crosslinking. Within the cell, the combination of drug molecules modulates the signaling pathway to inhibit the secretion of PGE2, thereby achieving anti-inflammatory efficacy.

Drug-loaded biomaterials have emerged as promising platforms for diabetic wound healing. Aspirin, as one of the most effective nonsteroidal anti-inflammatory drugs, inhibits the cyclooxygenase-2 (COX-2) enzyme. COX-2 is a known mediator of prostaglandin E2 (PGE2) secretion, which is involved in promoting inflammatory conditions.^37,38^ The alleviation of the early inflammatory phase through COX-2 and PGE2 inhibition significantly impacts the diabetic wound healing process.^39−43^ In parallel, local anesthetics (LAs) suppress the production of inflammatory mediators including nitric oxide, PGE2, tumor necrosis factor (TNF)-α, interleukin (IL)-6, and IL-1β.^44,45^ The clinical administration of LAs to diabetic foot ulcers has demonstrated dual benefits: significantly reducing wound pain during and after dressing changes while promoting wound healing through the inhibition of the inflammatory PGE2 pathway via TNF-α suppression.^46−53^ However, both therapeutic approaches face limitations. Conventional LA administration typically only provides 2–4 h of drug efficacy,^54^ and while the combination of lidocaine and ropivacaine shows improved postoperative analgesia compared to single compounds,^55−57^ the acidosis present in inflamed tissues can diminish LA efficacy by reducing their ability to penetrate cell membranes.^58,59^ To overcome these challenges, LAs loaded in hydrogel carriers have emerged as a promising strategy, achieving sustained drug release over extended periods while minimizing toxicity and more effectively inhibiting the expression of pro-inflammatory cytokines and pain-related molecules including COX-2 and PGE2.^45,60−62^ Therefore, the development of composite materials with comprehensive functions that enable the synergistic action of anti-inflammatory drugs and LAs is crucial for enhancing wound healing and improving the quality of life for diabetic patients.

In this work, we demonstrate a sustainable therapeutic platform comprising hydrogels enhanced with drug-loaded nanoparticles (DNPs) featuring well-defined porous structures for diabetic wound treatment (see Scheme 1). A cocktail combination of aspirin, ropivacaine, and lidocaine nanoparticles was developed to achieve synergistic therapeutic effects in suppressing both COX-2 and TNF-α pathways, thereby reducing the secretion of inflammatory and pain mediator PGE2. The drug nanoparticles were fabricated via a reprecipitation method and encapsulated with phospholipid-acrylate polymers. The surface acrylate-rich DNPs were cross-linked with polyethylene glycol diacrylate (PEGDA) hydrogels, forming distinct porous structures that further enhanced the sustained drug release capabilities. The localized hydrogel dressing, when applied to diabetic wounds, exhibited continuous release of the three therapeutic agents over a period ranging from 4 to 72 h. The nanodrug–hydrogel composite enabled localized treatment by mediating signaling pathways exclusively in wound site cells without affecting other regions. The hydrogel-treated wounds demonstrated a 17-fold increase in the skin healing rate compared to untreated wounds, promoting re-epithelialization, angiogenesis, and collagen regeneration while facilitating scarless healing of diabetic wounds concurrent with inflammation resolution.

## 2 Results and Discussion

### 2.1 Preparation and Characterizations of DNPs

**Figure 1:**
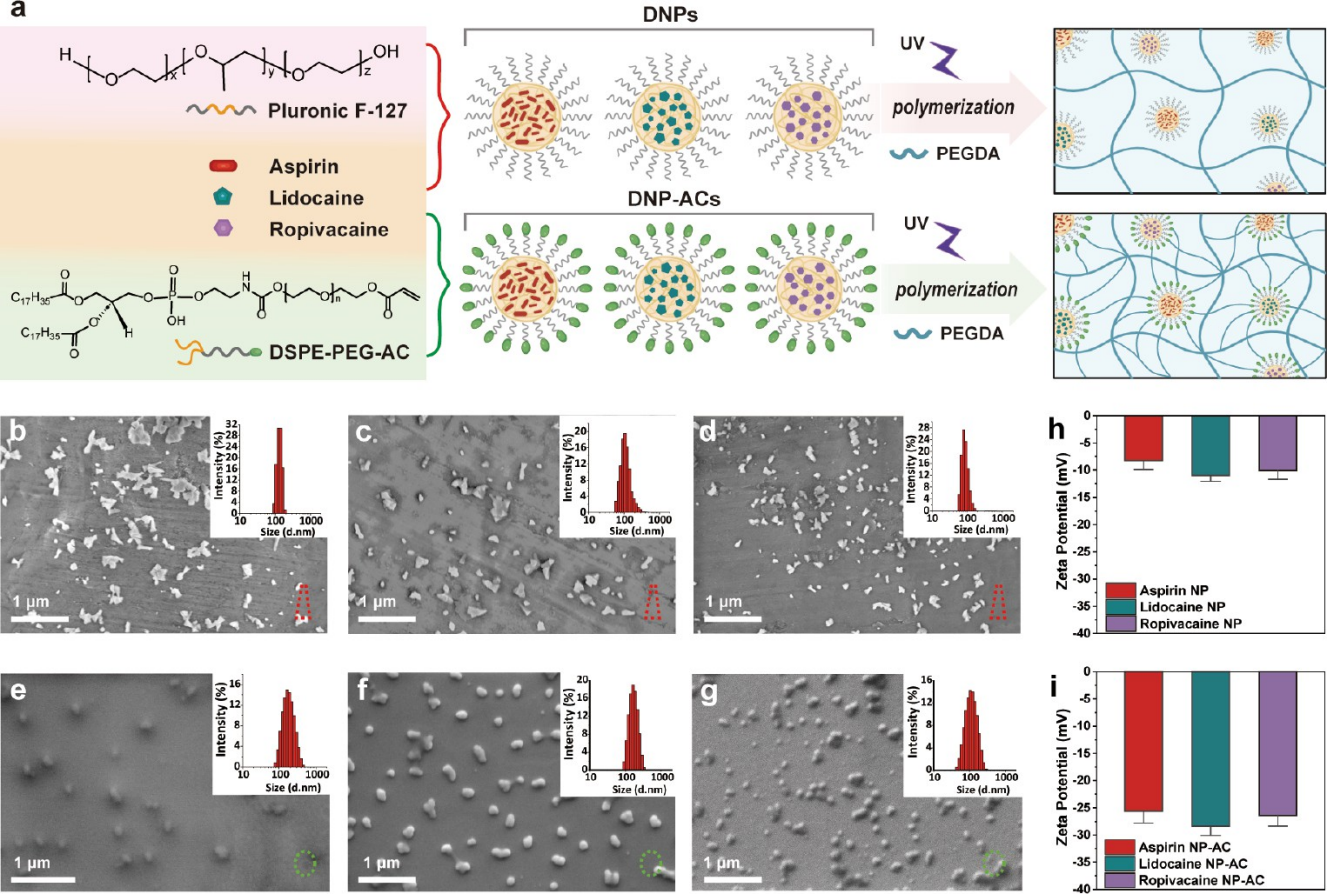
a) Scheme of DNPs and nanodrug–hydrogel composite preparation. (b–d) Typical SEM images and DLS size distribution of DNPs encapsulated by F-127 (aspirin, lidocaine, and ropivacaine from left to right). (e–g) Typical SEM images and DLS size distribution of DNP-ACs encapsulated by DSPE-PEG-AC (aspirin, lidocaine, and ropivacaine, from left to right). (h,i) Zeta potentials of DNPs without and with acrylates in water, respectively.

We developed a sustained delivery system that incorporates a cocktail formulation of anti-inflammatory and LA nanoparticles. As shown in Figure 1a, the drug nanoparticles were encapsulated by two types of amphiphilic polymers, respectively. Pluronic F-127 is a nonionic surfactant polyol that facilitates the solubilization of hydrophobic drugs in physiological media to assemble the DNPs. The amphiphilic polymer (DSPE-PEG-AC) is also a typical encapsulation agent but contains acrylate groups (green point) to assemble DNP-acrylates (DNP-ACs) that are cross-linkable with one of the gel precursors PEGDA. Subsequently, PEGDA can be cross-linked with DNP-ACs to reinforce the pore structure of hydrogel for long-lasting drug release.

We prepared aspirin, ropivacaine, and lidocaine nanoparticles by a reprecipitation method, where Pluronic F-127 or DSPE-PEG-AC was used to encapsulate the nanodrugs to achieve water solubility and stability. The aspirin, lidocaine, and ropivacaine DNPs encapsulated by Pluronic F-127 were characterized by SEM and DLS measurements (Figure 1b–d), indicating their particle diameters of 122, 105, and 79 nm, respectively. These nanoparticles exhibit irregular shapes and low values of zeta potentials (Figure 1h). By contrast, the aspirin, lidocaine, and ropivacaine DNP-ACs encapsulated with the DSPE-PEG-AC polymer showed regular spheroid shapes with the particle size of 175, 164, and 101 nm, respectively (Figure 1e–g). DSPE-PEG-AC provided a homogeneous assembly of DNP-ACs as well as an outer layer distribution of acrylates, contributing to zeta potentials that were approximately three times higher than those of DNPs without acrylates (Figure 1i). The larger value of the zeta potential led to a more stable distribution of drug nanoparticles in water. Meanwhile, the surface acrylates are cross-linkable with PEGDA, facilitating the stable loading of DNP-ACs in the hydrogel.

The UV–vis absorption spectra of all drug nanoparticles are demonstrated in Figure S1. Drug encapsulation efficiency (EE %) was determined via UV–vis spectroscopy and ultrafiltration centrifugation (Figure S2). The F-127-coated nanoparticles showed an EE % of 79.56 ± 0.25%, 82.59 ± 0.14%, and 77.47 ± 0.25% for aspirin, ropivacaine, and lidocaine, respectively. DSPE-PEG-AC-coated formulations demonstrated enhanced encapsulation, achieving 82.86 ± 0.18%, 85.29 ± 0.26%, and 87.30 ± 0.15% for the corresponding drugs. These results demonstrate that DSPE-PEG-AC coating not only provides surface functionality but also achieves higher drug EE % compared to F-127 coating across all three drug molecules.

### 2.2 Morphology Characterizations and Drug Release Properties of the Drug-Loaded Hydrogel

**Figure 2:**
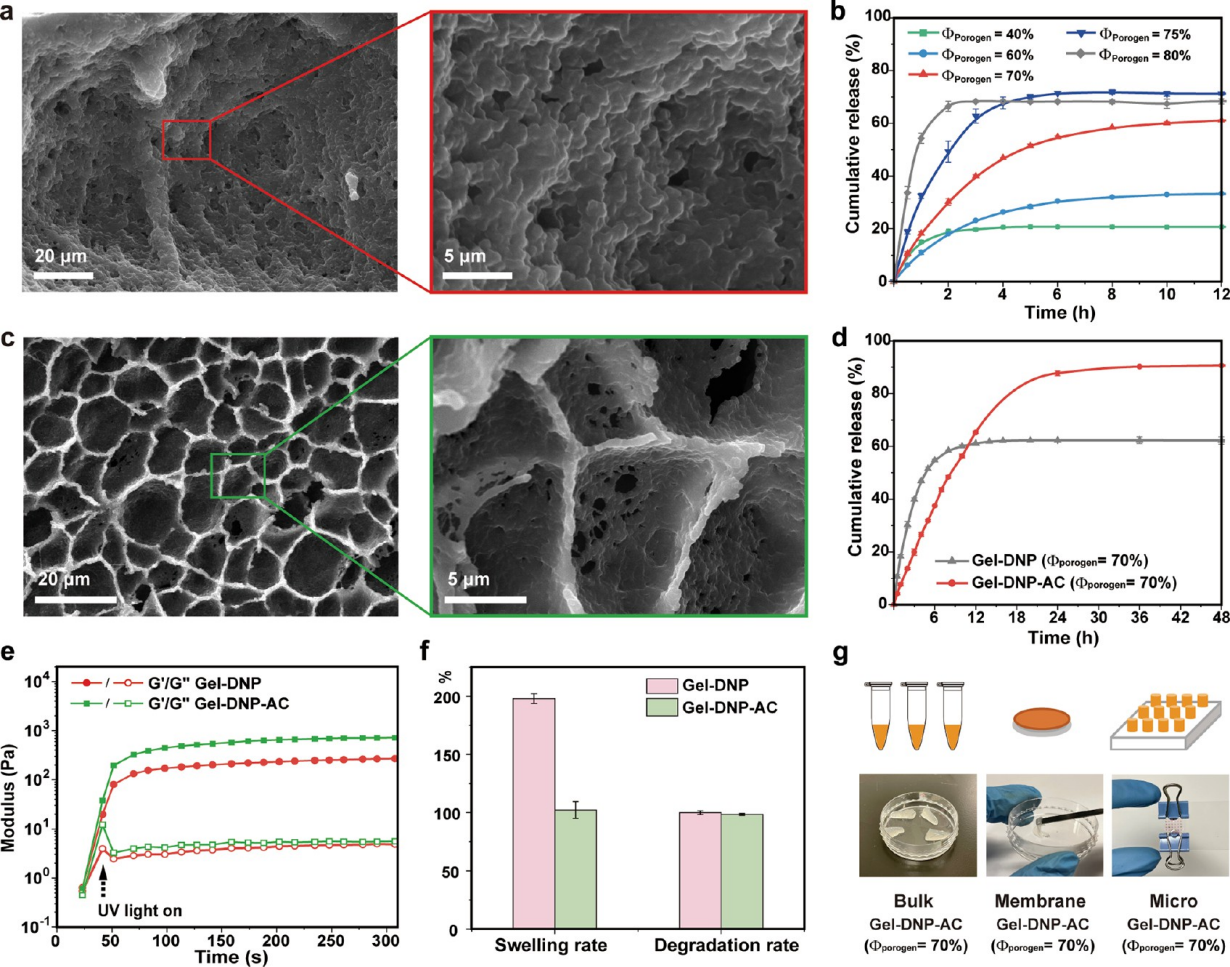
(a) Typical SEM image of Gel-DNP. (b) Cumulative release curves of bulk Gel-DNP with different porogen fractions from 40% to 80%. (c) Typical SEM image of Gel-DNP-AC. (d) Cumulative release curves of bulk Gel-DNP and Gel-DNP-AC with a porogen fraction of 70%. (e) Rheological profiles showing hydrogel formation by UV light-induced photopolymerization of Gel-DNP and Gel-DNP-AC, respectively. (f) Swelling and degradation properties of two kinds of the hydrogel. Swelling rates of the hydrogels measured in deionized water at room temperature until reaching equilibrium weight, and degradation rates (DRs) of the hydrogels measured in deionized water for 14 days. Data are presented as mean ± SD (*n* = 3). (g) Illustration of different shapes of the hydrogel: bulk, membrane, and micro of Gel-DNP-AC with a porogen fraction of 70%.

Drug-loaded hydrogels were prepared by cross-linkable PEGDA, porogenic polyethylene glycol (PEG), and DNP solution. We used PEGDA and PEG to form the hydrogel because they have demonstrated to have good biocompatibility, biodegradability, and immunologically inertness.^63,64^ PEG has been used as a porogen to introduce porosity in the hydrogel for drug-loading accessibility.^65^Figure 2a shows the morphology of the hydrogels loaded with the DNPs (Gel-DNP), which is prepared by using the precursor formulation with PEGDA 10 wt %, PEG 70 wt %, and DNP solution 17.6 wt %. The PEG porogen provides the structure of the hydrogel with channels resembling caves, facilitating the loading of DNP solutions. Therefore, the amount of porogen affected the drug release properties of the hydrogel. We investigated the effect of the porogenic PEG component on the drug release properties by placing the Gel-DNP in Phosphate Buffered Saline (PBS) solution rocked at a constant temperature of 37 °C. The hydrogels were prepared by varying the porogenic PEG fraction from 20 to 90 wt %, while the DNP concentration remained constant. The detailed compositions of the precursor are listed in Table S1. We observed that the release time was prolonged with an increase in the porogen fraction before Φ_porogen_ = 70% (Figure 2b). At 70% PEG fraction, the complete release time of the drug reached up to 12 h. However, the increase in porosity was accompanied by a decrease in the structural strength. Beyond the optimal point Φ_porogen_ = 70%, the complete release time began to decrease; for instance, the complete release time was 6 h for Φ_porogen_ = 75%. It was noted that the Gel-DNP with Φ_porogen_ = 80% disintegrated after 2 h in an in vivo environmental simulation (Figure S3). The excessive addition of the porogen ratio leads to an increase in the surface area and structural instability of the hydrogels. This causes the hydrogel to collapse quickly in body fluids, resulting in a rapid release of the drug. To maintain steady-state concentration, the formulation of the gel precursor was controlled at Φ_porogen_ = 70%.

The surface acylate groups of the nanodrugs are essential for the formation of regular pore structures in the hydrogel. We formed the hydrogels cross-linked with DNP-ACs (Gel-DNP-AC) from the precursor consisting of PEGDA 10 wt %, PEG 70 wt %, and DNP-AC solution 17.5 wt %. As shown in Figure 2c, the Gel-DNP-AC platform shows the well-defined pore structures compared to the Gel-DNP, indicating the essential role of DNP-AC in the formation of regular pores. Magnified SEM images show that the pore diameters in Gel-DNP-AC vary in the range of a few tens of microns. The regular pores and channels facilitate drug release from the Gel-DNP-AC hydrogel. We investigated the effect of cross-linking between nanodrugs and hydrogels on drug release properties by comparing Gel-DNP and Gel-DNP-AC at the same PEG fraction (Φ_porogen_ = 70%). It is evident that Gel-DNP-AC exhibited a sustainable release performance. The Gel-DNP-AC showed a release time more than 24 h and a cumulative drug release quantity higher than 90% (Figure 2d), indicating that the cross-linking between DNP-ACs and hydrogel results in a long-sustainable and complete release compared to the gel physically loaded with DNPs. Cross-linking between DNP-ACs and PEGDA likely creates a large number of stable attachment sites for nanodrugs on the hydrogel scaffold, enhancing the drug-loading capacity of the composite hydrogel.

The dynamic rheological properties were analyzed through a time sweep test (Figure 2e). The storage modulus (*G*′) was larger than the loss modulus (*G*″) after light treatment. Both gel precursors with two types of DNPs turned from liquid to solid in a few seconds upon UV-light illumination and exhibited elastic behavior immediately. The storage modulus of Gel-DNP-AC was larger than that of Gel-DNP, indicating that the involvement of DNP-ACs in the hydrogel framework enhanced the mechanical properties of the hydrogels. The improved mechanical properties are consistent with the well-defined pore structures of the Gel-DNP-AC sample. FTIR spectroscopic analysis was performed to verify the polymerization of the Gel-DNP and Gel-DNP-AC systems. The characteristic shift of carbonyl bands in the FTIR spectra (Figure S4) provided evidence for successful polymerization, demonstrating the transformation from prepolymers to their respective hydrogel networks. The swelling and DRs of two structured hydrogels were evaluated in PBS buffer (pH 7.4). As shown in Figure 2f, both hydrogels were degraded by up to 97% after 1 week of soaking in PBS, indicating the hydrogels are well biodegradable. The water uptake capacity of the Gel-DNP-AC was significantly lower than that of the Gel-DNP, demonstrating the rigid framework with a pore structure in Gel-DNP-AC. The acrylate’s surface modification on DNP-ACs increases the cross-linking density of the composite hydrogel and leads to the formation of regular pore structures, thus limiting the swelling properties of the hydrogel. The swelling properties of hydrogels are highly indicative of the absorption of the wound exudate of the wound dressing. A low swelling rate of the hydrogels is conducive to maintaining the mechanical strength for dermal therapeutic applications. We also investigated the influence of the hydrogel shape on drug release. As shown in Figure 2g, the precursor solutions of 70% porogen and DNP-ACs were added to molds and cured under UV light to form bulk hydrogel, membrane hydrogel, and micro hydrogel. The microgel had the slowest release rates among the three shapes, which might be related to the larger specific surface area of the microgel and the different mechanical properties of oscillating in the fluid. The membrane hydrogel, suitable for skin treatments, exhibits release properties comparable to those of the bulk hydrogel, with a complete release time shorter than 24 h (Figure S5).

**Figure 3:**
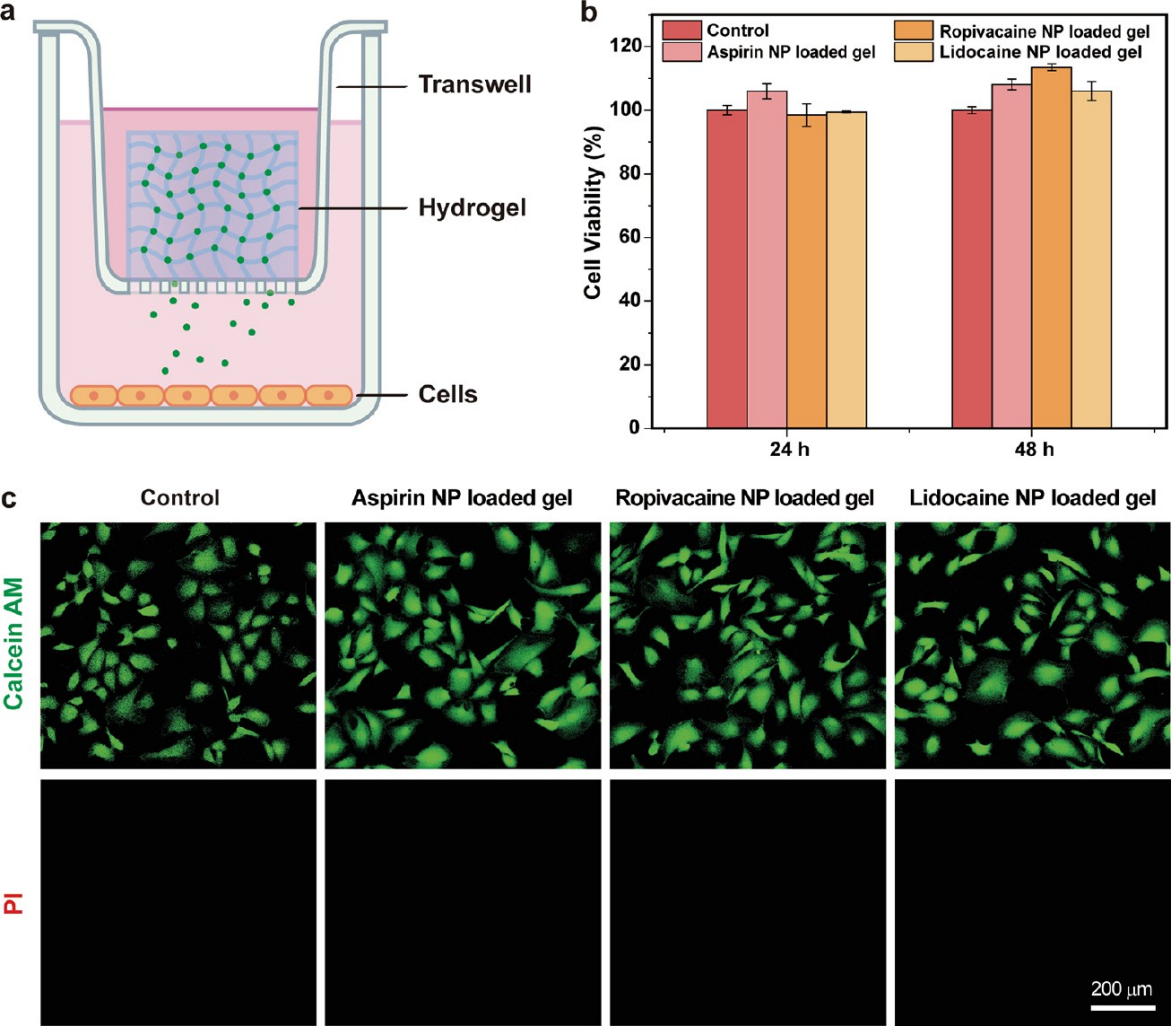
(a) Schematic illustration of biocompatibility tests of hydrogels with mammalian cells via transwell. (b) Viability of B-SC-1 cells treated with Gel-DNP-AC for 24 and 48 h. Data are reported as mean ± SD (*n* = 5). (c) Fluorescence imaging of Live/Dead kit-stained B-SC-1 cells treated with Gel-DNP-AC for 48 h, where calcein AM (green) stained live cells and PI (red) stained dead cells. Scale bar: 200 μm.

We examined the cytotoxicity of Gel-DNP-AC by using hemolysis and a transwell assay. The hemolysis results using rabbit blood indicated good compatibility of the hydrogel scaffold with blood cells (Figure S6). The biocompatibility of Gel-DNP-AC was further evaluated by MTT and cell staining assays. Three Gel-DNP-AC gels were investigated using the transwell device to evaluate the release of drugs and leaching of monomer residues. Mammalian cells (BS-C-1) were treated with the hydrogels placed in the transwell inserts (Figure 3a). All Gel-DNP-AC exhibit no significant cytotoxicity, as indicated by the approximately 100% cell viability after 48 h of incubation (Figure 3b). Fluorescence staining displayed that the cells treated with the hydrogels containing the three types of DNP-ACs were similar to the control group (Figure 3c), as evidenced by the bright fluorescence of live cells (calcein AM) and the absence of fluorescence from dead cells (PI). These observations further confirmed the good biocompatibility of Gel-DNP-AC.

### 2.3 Diabetic Wound Healing by the Drug-Loaded Hydrogel

Scratch assays were performed on both human keratinocytes (HaCaT) and human umbilical vein endothelial cells (HUVECs) to evaluate cell migration (Figure S7). We compared the migration-promoting effects of hydrogels loaded with individual DNP-AC and the triple-DNP-loaded hydrogel system (Gel-DNP-AC). Among single-DNP formulations, ropivacaine NP-loaded hydrogels showed superior promotion of HaCaT migration, while aspirin NP-loaded hydrogels demonstrated the highest effect on HUVEC migration. This cell-specific response suggests differential drug uptake and utilization mechanisms, highlighting the importance of multidrug combinations in wound healing. Notably, Gel-DNP-AC demonstrated the highest promotion of cell migration in both cell types, indicating a synergistic effect of the combined delivery system for enhanced wound healing through multiple cellular pathways.

**Figure 4:**
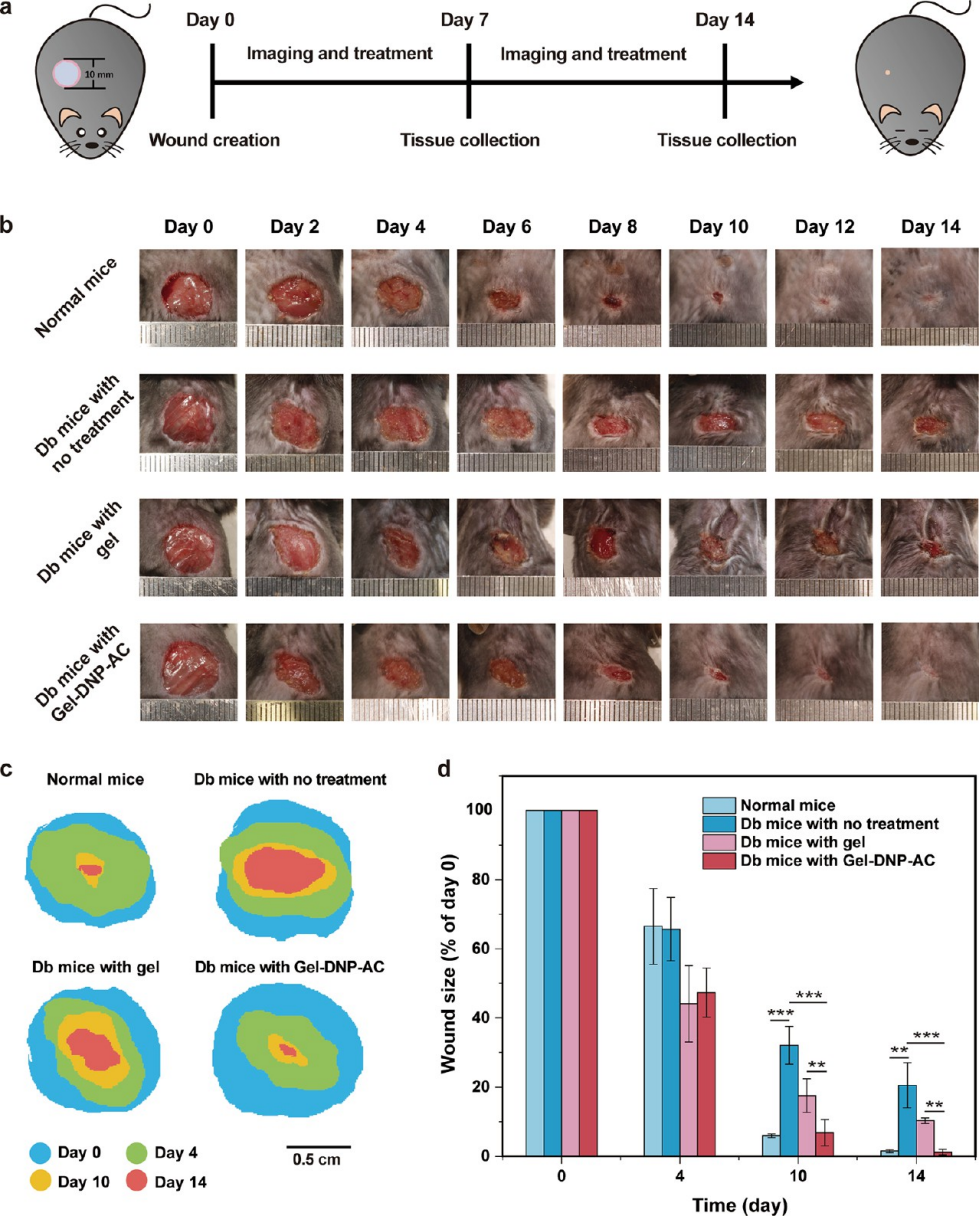
(a) Schematic illustration of the design of animal experiments to test the therapeutic effect of the composite hydrogel in the normal and diabetic mouse model. (b) Representative images of the wounds treated with or without the composite hydrogel for 14 days. (c) Illustration of wound size at different time points, normalized to day 0. (d) Qualification of wound size change during 14 days of post wounding. *P < 0.05, **P < 0.01, and ***P < 0.001, *n* = 4.

We explore diabetic wound healing by pasting the Gel-DNP-AC gel patch on the excisional wounds of diabetic mice (Figure 4a). The anti-inflammatory DNP-ACs and anesthetic DNP-ACs were loaded into the composite hydrogel via cross-linking for a cocktail therapy. The therapeutic efficacy of the drug nanoparticles was evaluated using lipopolysaccharide (LPS)-induced RAW 264.7 macrophages. The combined treatment with DNPs demonstrated maximum reduction in the secretion of inflammatory mediators, including TNF-α, NF-κB, and PGE2 (Figure S8), validating the cocktail combination’s effectiveness in inflammatory pathway suppression. The dosage of the cocktail combination consists of 40 μg of aspirin, 30 μg of ropivacaine, and 30 μg of lidocaine for each gel patch. As shown in Figure 4b, the wounds of diabetic (Db) mice treated with the Gel-DNP-AC exhibited a higher closure rate than those with no treatment and the pure gel group. By day 14, the wound of diabetic mice treated with the Gel-DNP-AC group is completely closed, as compared to the control groups (Figure 4d). Body weight analysis throughout the experiment indicated that the Gel-DNP-AC hydrogel system had no adverse effects on the health of mice (Figure S9). The wound healing achieved 1.16% closure relative to the original size in the Gel-DNP-AC treatment group, exhibiting similar or better wound closure rates compared with previous studies that used anti-inflammatory drugs to treat chronic wounds in the same rodent model (STZ-induced diabetic mouse).^3,17,66,67^ The wound area of the Gel-DNP-AC group closed quicker than the normal mice group in the initial 4 days (Figure 4c). These results indicated that the porous hydrogel promoted wound closure in the initial period and the Gel-DNP-AC helps diabetic wounds recover as effectively as normal ones.

**Figure 5:**
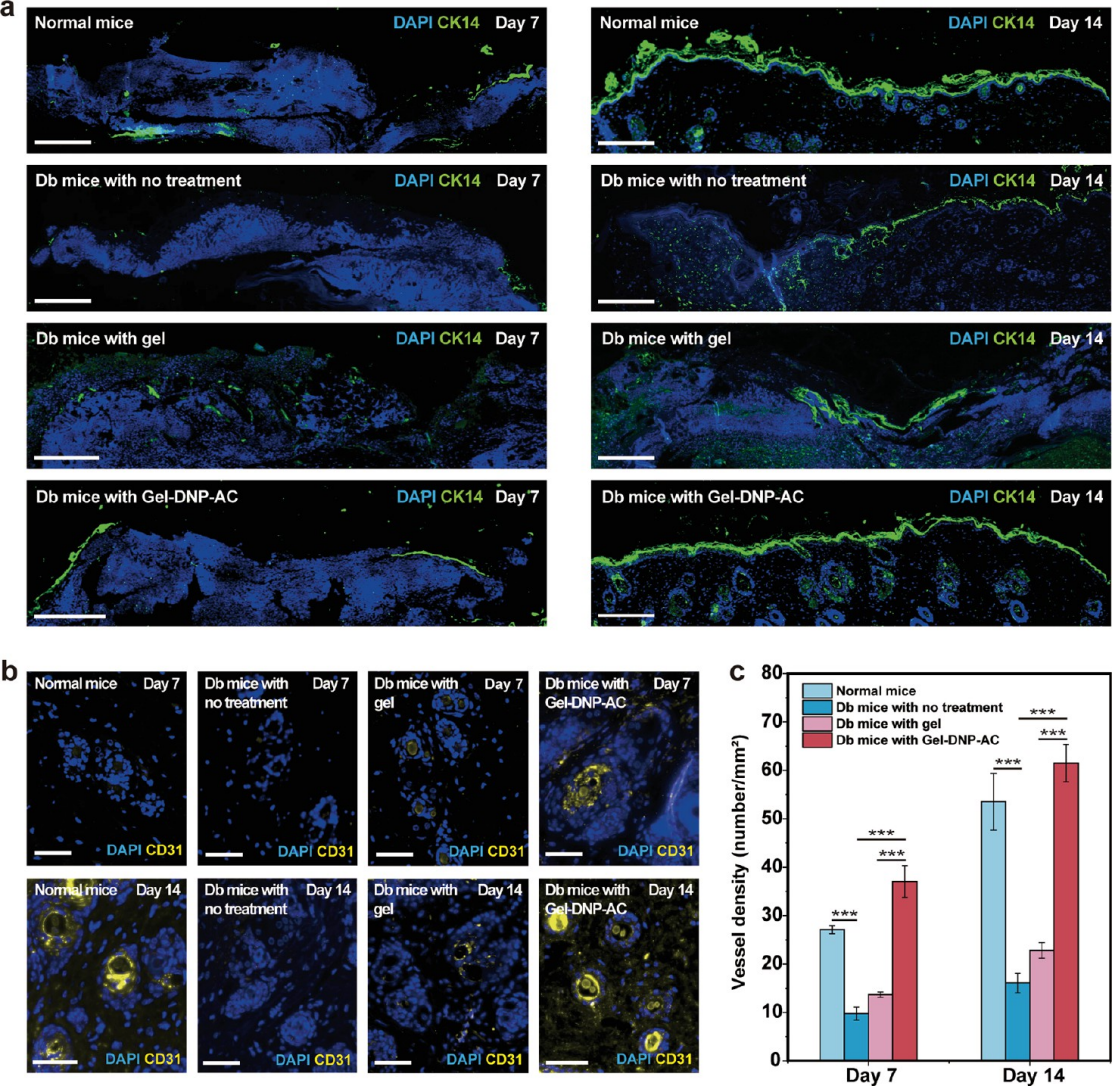
(a) Immunofluorescence staining of cytokeratin 14 (CK14, red) for the wounds on days 7 and 14. Scale bars, 200 μm. (b) Immunofluorescence staining of vessel (CD31, yellow) in the wounded region at 7 and 14 days. Scale bars, 50 μm. Nuclei were stained with DAPI in all immunofluorescence staining images. (c) Quantification of vessel density. *P < 0.05, **P < 0.01, and ***P < 0.001, *n* = 4.

We evaluated the rate of re-epithelialization in diabetic wound sites by using cytokeratin 14 (CK14, a marker for basal keratinocytes) to stain tissue sections collected on days 7 and 14. On day 7, the midpoint of the cure period, the wound gap was half enclosed by the layer of basal keratinocytes (CK14^+^) in the Gel-DNP-AC group. In contrast, keratinocyte migration in the other control groups was obviously slower than that in the treatment group (Figure 5a). On day 14, the migration of basal keratinocytes in the Gel-DNP-AC group and the normal mice group was fully complete. In the Gel-DNP-AC group, it had a clearer stratified epithelium as compared to the normal mice without gel. By contrast, in the diabetic mice with no treatment group and treated with the gel alone without drug loading, the keratinocyte layers were unclosed, while the migration, proliferation, and differentiation were still ongoing. These observations prove that the continuous anti-inflammation together with pain relief contributed by Gel-DNP-AC effectively accelerated keratinocyte migration, consequently promoting re-epithelialization of the diabetic wounds.

To evaluate the angiogenesis in the diabetic wounds, we also used a platelet endothelial cell adhesion molecule-1 (CD31) marker to quantify the capillary densities at the middle stage (day 7) and final stage (day 14) of wound healing. At both stages, the Gel-DNP-AC group had a significantly higher density of capillaries (CD31^+^) than other control groups (Figure 5b,c, *P* < 0.001). The results demonstrate that the composite Gel-DNP-AC facilitates rapid angiogenesis and assists the chronic wound in transitioning from the prolonged inflammation phase to the proliferation phase.

**Figure 6:**
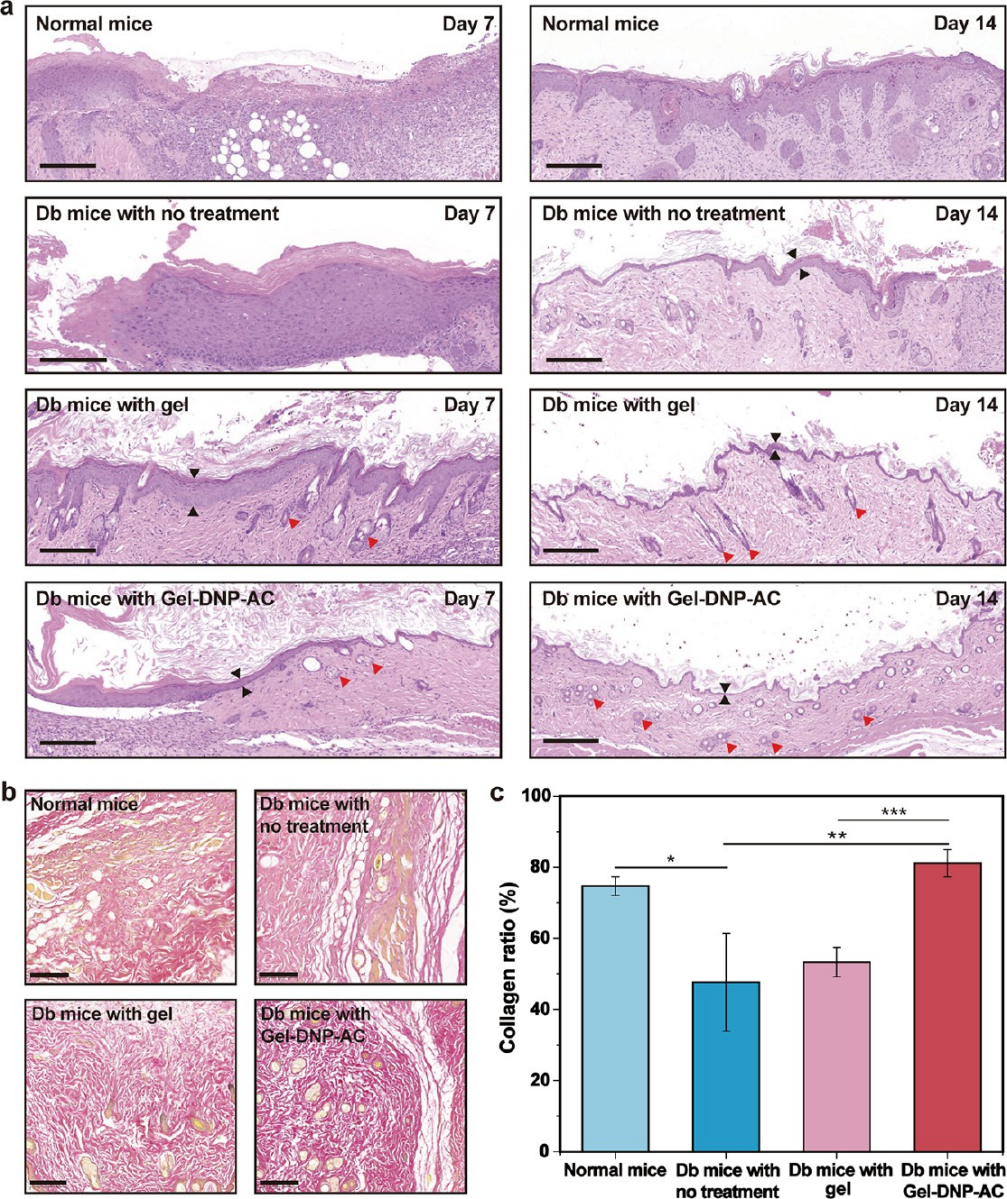
Drug-loaded hydrogel promoting scarless wound healing in diabetic wounds. (a) H&E staining images of wounds after 7 and 14 days. Scale bar, 200 μm (black arrow, epidermis; red arrow, skin appendages). (b) Picrosirius red staining of the wounded skin at day 14. Scale bar, 100 μm. (c) Quantification of total collagen deposition at day 14. *P < 0.05, **P < 0.01, and ***P < 0.001, *n* = 4.

To evaluate the scarless skin repair in the diabetic wound sites, we then characterized the epidermal thickness by hematoxylin–eosin (H&E) staining. Inflammatory cellular status, fibroblast proliferation, and neovascularization of the wound were demonstrated by H&E staining (Figure 6a). On day 7, both the normal mice group and diabetic mice with no treatment exhibited a large number of inflammatory cell infiltration. By contrast, the pure gel group and the Gel-DNP-AC group exhibited fewer inflammatory cells, and some skin appendages (red arrow) were regenerated, indicating that PEGDA hydrogels facilitate the prompt functional rehabilitation of the skin tissues. On day 14, a large number of skin appendages and hair follicles had formed in the hydrogel-treatment groups, whereas much less were observed in other control groups. Among the four groups, the Gel-DNP-AC group exhibited the most effective regeneration, with a regular arrangement of collagen fibers and skin appendages as well as the thinnest neoepidermis (black arrow) compared to all other control groups. This indicates that the nanodrug-hydrogel composite sustainable therapeutic platform can regulate the infiltration of inflammatory cells in diabetic wounds, promoting the regeneration of skin tissues and the proliferation of fibroblasts for scarless skin.

Finally, we evaluated collagen regeneration in the Gel-DNP-AC treatment, which is a key indicator of wound healing. Sirius scarlet dye stains collagen into red and other tissues into yellow. We compared the density and depth of the two colors to determine the collagen content at the wound site. As shown in Figure 6, a large amount of collagen with a high density of red area was regenerated at the trauma site in the Gel-DNP-AC group. On day 14, there was less red-colored collagen in the three control groups compared to the Gel-DNP-AC group (81% of the total healing area, *P* < 0.01) (Figure 6b,c). The collagen regeneration was neatly aligned and flat, revealing that our enhanced wound treatment method contributes to scarless wound healing.

In summary, the superior wound healing performance of the Gel-DNP-AC system can be attributed to the synergistic effects of sustained drug release and the optimal microenvironment provided by the hydrogel matrix. The comprehensive histological analyses revealed accelerated re-epithelialization with enhanced CK14^+^ keratinocyte migration and increased CD31^+^ capillary density at day 7, while the well-organized collagen deposition and thin neoepidermis at day 14 indicated a more physiologically favorable healing process. The reduced inflammatory cell infiltration and enhanced skin appendage regeneration, as evidenced by H&E staining, further validated that our nanodrug–hydrogel composite system effectively modulates the inflammatory microenvironment while promoting tissue reconstruction. These findings collectively demonstrate that the engineered delivery platform achieves optimal therapeutic outcomes through maintaining sustained drug release and creating a conducive healing microenvironment. Nevertheless, there remains challenges to be addressed in future investigations. Comprehensive pharmacokinetic studies will further reveal the systemic effects of prolonged material exposure. Furthermore, while our current platform demonstrates promising controlled release capabilities, future developments of smart stimulus-responsive elements and multicompartment architectures will enable more precise control over drug release profiles, advancing the platform toward intelligent wound healing applications.

## 3 Conclusions

We developed a nanoparticle–hydrogel composite platform with robust sustained-release and immunoregulatory properties for chronic diabetic wounds. The hydrogel patch consisted of a cocktail combination of cross-linkable DNP-AC, UV-induced cross-linked PEGDA, and porous component PEG. The fraction of hydrogel components was adjusted for optimal release property. Phospholipid-acrylate polymer was used to modify the DNP-AC surface that can cross-link with the hydrogel matrix for structure enhancement. The well-defined pore structure improved the sustained release of the drug-loaded hydrogel. The Gel-DNP-AC showed effective anti-inflammatory activity by augmenting the survival and migration of keratinocytes and facilitating the neovascularization and collagen alignment in diabetic wounds. Our results demonstrate an effective therapeutic outcome for the accelerated healing of diabetic wounds using the hydrogel patch. Beyond diabetic wounds, it may also be used in postsurgical wounds for long-lasting anti-inflammatory and analgesic applications. The designable shape gives it a high potential to be applied to skin wounds and surgical sutures as a drug reservoir, and its sustained-release properties eliminate the need for repeated dressing changes.

## 4 Experimental Section

### 4.1 Materials

All chemicals were purchased from Sigma-Aldrich (Hong Kong S.A.R., China) unless otherwise stated. All materials were used without further purification, unless otherwise stated. PEGDA (Mn = 700), PEG (Mn = 600), and 2-Hydroxy-2-methylpropiophenone (Darocur 1173) were used for hydrogel cross-linking. Aspirin, lidocaine, ropivacaine, Pluronic F-127, and DSPE-PEG-AC (obtained from Biopharma PEG Scientific Inc. (USA)) were used for drug nanoparticle encapsulation. Tetrahydrofuran (THF) was pretreated with sodium, followed by distillation for the purpose of preparing the drug nanoparticles. PBS was used to simulate osmotic pressure and test drug release curve in vitro. Ultrapure H_2_O (18.25 MΩ cm^–2^ at 25 °C) was used throughout the study, and all other chemical reagents were used as received.

### 4.2 Fabrication of DNPs with Surface Function

Anti-inflammatory/LA drug nanoparticles were fabricated by using the reprecipitation method. The organic drug molecule was dissolved with a concentration of 1000 ppm in THF, respectively. The polymer coating agents were dissolved in THF at the same concentration. The mixture of 200 μL of the drug solvent (1000 ppm) and 200 μL of the polymer solvent (1000 ppm) was quickly dispersed into 10 mL of ultrapure water under ultrasonication. The THF was then removed by nitrogen bubbling. A small fraction of aggregates were removed by filtration through the 0.22 μm membrane filters.

### 4.3 Synthesis of Hydrogels

The PEGDA hydrogels were prepared by in situ free radical polymerization. The prepolymer solution of the hydrogel with different porosities, containing PEG at 40%, 60%, 70%, 72%, 75%, and 80% ratios of total weight and 33% PEGDA, 59% DI water or DNP solution, and 8% inhibitor of the excluded weight, was added into molds and cured under a UV lamp for 1 min. The obtained hydrogels were soaked in PBS to remove unreacted monomers and cross-linkers and freeze-dried overnight. The detailed compositions of all hydrogels are listed in Table S1. The hydrogels were soaked in PBS to remove unreacted monomers and cross-linkers. The concentrated drug nanoparticle (encapsulated by F-127 or DSPE-PEG-AC) solution was used to replace the aqueous phase in the prepolymer as described above to synthesize the more robust frameworks of DNP-loaded hydrogels.

### 4.4 Morphology of DNPs and Hydrogels Characterized by SEM

The DNP solution was dripped on the silicon wafer surface and then attached on the copper platform after evaporating to dryness and subsequently coated with carbon to increase inductivity of the organic particles. PEGDA hydrogel was freeze-dried as the sample and tore apart by forceps, revealing the morphology of their side-section surfaces. The hydrogel samples were fixed on the copper platform and subsequently coated with gold to increase inductivity before observation.

### 4.5 Swelling Rate and Dynamic Rheological Test of Hydrogels

The weight method was used to determine the swelling rate of the hydrogels in water. The hydrogel samples were soaked in water until they reached a constant weight and then were quickly removed from the water. After the removal of water from the hydrogel surface with filter paper, the samples were weighed to obtain the equilibrium swelling mass. The equilibrium swelling rate (ESR) of the hydrogel was calculated by the following equation
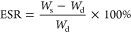
where *W*_s_ is the weight of the hydrogel after dissolution equilibrium; and *W*_d_ is the dry weight of the hydrogel (lyophilized).

The degradation rate (DR) was evaluated by the weight of the samples that underwent freeze-drying after swelling immersion. The weight method was used to determine the DR of the hydrogels in water. The hydrogel samples were soaked in water until they reached a constant weight and then were removed from the water and freeze-dried. After removal of water from the hydrogel, the samples were weighed to obtain the degraded mass. The DR of the hydrogel was calculated by the following equation
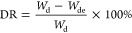
where *W*_d_ is the dry weight of the hydrogel before immersion; and *W*_de_ is the dry weight of the hydrogel after immersion.

The dynamic rheological properties of hydrogels were tested by a rotational rheometer (HAAKE MARS III). With UV-light illumination, the storage modulus and loss modulus were measured with the time and frequency change.

### 4.6 Sustained Release Test of Hydrogels

The DNP-loaded hydrogels were placed into PBS in flat-bottom centrifuge tubes and kept at 37 °C in the incubator with a shaker at 60 rpm to mimic in vivo environment. One mL of the drug release solution was periodically pipetted from the centrifuge tube for UV–vis testing. PBS was replaced at every interval with the same volume of fresh PBS to maintain perfect sink condition. The immersion solution was analyzed by a UV–vis spectrophotometer at the drug-characterized absorption peak wavelength. The cumulative release absorption of the hydrogels was calculated as follows
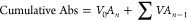
where *A*_*n*_ and *A*_*n*-1_ are the drug absorption for sampling for *n* times and *n*-1 times, respectively, *V*_0_ is the initial volume of the drug release medium, and *V* is the sampling volume.

### 4.7 Cell Culture and Cytotoxicity Assays

BS-C-1 cells were cultured at 37 °C in Dulbecco’s modified Eagle’s medium containing 10% fetal bovine serum and 1% penicillin/streptomycin with a humidified environment containing 5% CO_2_. The cell activities and cytotoxicity of Gel-DNP-AC in vitro were evaluated using MTT and calcein AM assays. BS-C-1 cells with a total number of 1 × 10^4^ cells per well were seeded into 24-well cell culture plates until adherent overnight. Then, the hydrogels were cut into cylindrical shapes (6 mm* 2 mm) and put in the transwell chamber, and the chamber was sequentially placed on the top of cells, soaking hydrogels for drug release. The cells were incubated at 37 °C in an atmosphere containing 5% CO_2_ for 24 or 48 h. After incubation, MTT was added to each well, and samples were incubated for an additional 4 h. After the addition of dimethyl sulfoxide, a microplate reader (BioTek Cytation 3) was used to measure the absorbance value (OD570) of each well with background subtraction. The viability of cells was calculated according to the following equation
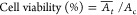
where  is the mean absorbance of the treatment group, and *A*_c_ is the mean absorbance of the control group.

The cytocompatibility of Gel-DNP-AC was tested with the Live/Dead cell viability kit. The cells were seeded and incubated with the hydrogel soaking in a transwell as above. Then, the calcein AM solution and PI solution were added to each well for 30 and 10 min of incubation, respectively. The samples were washed with PBS three times and observed by confocal microscopy.

### 4.8 Diabetic Mice Induction and Wound Treatment

The diabetic mice were induced by the Streptozotocin (STZ) method. Male C57BL/6J mice (20–25 g) were purchased from Baineng Biotechnology Co., Ltd. (Guangzhou, China). All experiments were conducted in strict accordance with international ethical guidelines, following the relevant ethical policies and requirements of the Ethics Committee at the Southern University of Science and Technology (certificate SUSTC-JY202310106). Throughout the experimental procedures, we have made efforts to minimize the number of animals used and alleviate any potential pain or discomfort. STZ was dissolved in a citric acid and sodium citrate buffer. Each mouse was administered with an intraperitoneal injection of STZ (180 mg/kg) to induce diabetes, and the administration continued until stable blood glucose levels exceeded 20 mmol/L after 10 days. Blood glucose level was measured for the mice with overnight fasting. For each normal and diabetic mouse, a circular wound with a 1 cm diameter was created on the depilated dorsal fur for subsequent treatments. Diabetic mice were randomly divided into three groups (*n* = 5). We performed treatment with the blank hydrogel and Gel-DNP-AC starting on days 0, 2, 4, 6, 8, and 10 postwound establishment. Mice were euthanized on days 7 and 14, and skin tissues from the wound site on the back were collected. Tissues were fixed in 4% paraformaldehyde, embedded in paraffin, and subjected to histological staining with H&E staining, Picrosirius red staining, and immunofluorescent staining of CK14 and CD31.

## Supporting Information Available

UV–vis spectra of all drug nanoparticles; standard concentration curves for aspirin, ropivacaine, and lidocaine established by UV–vis spectrophotometry; reactant dosage of Gel-DNP prepolymer solution; FTIR spectrum of the prepolymer materials (PEG and PEGDA), amphiphilic polymers (F-127 and DSPE-PEG-AC), and both types of hydrogel systems postpolymerization; shape of hydrogels with different PEG ratios before and after soaking; cumulative release curves of different shapes of hydrogel; hemolysis rate of the Gel-DNP-AC; scratch assays evaluating cell migration; body weight record of mice during the experiment; antibacterial activity of hydrogels against *Escherichia coli* and *Staphylococcus aureus*; and expression of inflammatory mediators in LPS-stimulated RAW264.7 macrophages (PDF)
