# Supplementary material for: Nanoparticle-Reinforced Hydrogel with a Well-Defined Pore Structure for Sustainable Drug Release and Effective Wound Healing
Source: ACS Appl Bio Mater. 2025 Feb 7;8(2):1406–17. doi: 10.1021/acsabm.4c01659 (PMC11836925; doi:10.1021/acsabm.4c01659)
Supplement: Supplementary file 1 — mt4c01659_si_001.pdf [file mt4c01659_si_001.pdf]

Supporting Information

Nanoparticle-Reinforced Hydrogel with a Well-Defined Pore Structure for Sustainable Drug Release and Effective Wound Healing

Ziyi Zhang [a,b], Siyu Yang [c], Feixue Mi [d], Yicheng Yang [d], Qi Song [e], Yibo Gao [e], Changfeng Wu [d] and Weijia Wen [a,b,c]**

[a] Division of Emerging Interdisciplinary Areas, The Hong Kong University of Science and Technology, Clear Water Bay, Kowloon, Hong Kong 000000, China

[b] Thrust of Advanced Materials, The Hong Kong University of Science and Technology (Guangzhou), Nansha, Guangzhou 511400, China

[c] Department of Physics, The Hong Kong University of Science and Technology, Clear Water Bay, Kowloon, Hong Kong 000000, China

[d] Department of Biomedical Engineering, Southern University of Science and Technology, Shenzhen 518055, China

[e] Shenzhen Shineway Technology Corporation, Shenzhen 518048, China

**Correspondence and request for materials should be addressed to e-mail: phwen@ust.hk; wucf@sustech.edu.cn*

Methods

Encapsulation Efficiency Analysis: The concentration of drug nanoparticles (DNPs) was determined using a SHIMADZU UV-2600 spectrophotometer. Standard calibration curves were established using tetrahydrofuran (THF) solutions containing various concentrations of drug molecules. The encapsulation efficiency was calculated by analyzing the supernatant obtained after ultracentrifugation of the nanoparticle suspension (10,000 rpm, 20 min) using the precipitation method.

Fourier Transform Infrared Spectroscopy (FTIR): FTIR spectra were recorded on a Thermo Fisher Nicolet iS50 spectrometer with a resolution of 4 cm^{-1} and 32 scans per spectrum. Prior to analysis, hydrogel samples were subjected to overnight vacuum drying to eliminate moisture interference.

Cell Migration Assay: The wound healing ability was evaluated using a scratch assay. Briefly, cells were seeded in 12-well plates and allowed to reach confluence overnight. After creating uniform scratches using a cell scratcher, the cells were treated with hydrogel extracts from different groups. Cell migration was monitored and photographed using microscopy over a 24 h period.

Inflammatory Response Analysis: RAW264.7 macrophages were stimulated with lipopolysaccharide (LPS) and simultaneously treated with different DNPs formulations. After 4 h of incubation, both cells and culture supernatants were harvested for Western blot and enzyme-linked immunosorbent assay (ELISA) analyses to evaluate the expression and secretion of inflammatory mediators.

Antimicrobial Activity Assessment: The antimicrobial efficacy was evaluated using a colony forming unit (CFU) counting method. Bacterial suspensions were inoculated into fresh liquid medium containing sterilized hydrogel samples. After overnight incubation at 37 °C, the bacterial suspensions were appropriately diluted and plated on solid medium. CFUs were counted following overnight incubation to quantify the antimicrobial activity.

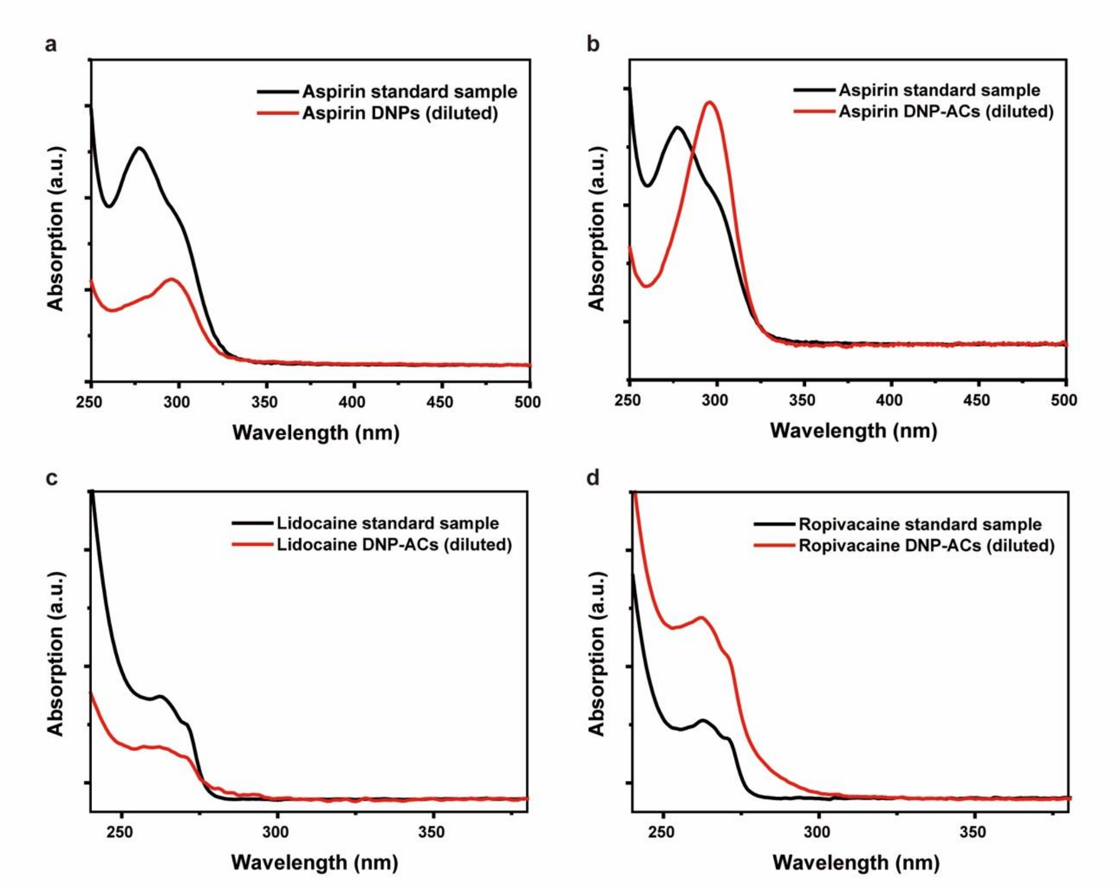

Figure S1. UV-Vis spectra of drug nanoparticles. a) aspirin NPs (F127), b) aspirin NPs (DSPE-PEG-AC), c) lidocaine NPs (DSPE-PEG-AC), d) ropivacaine NPs (DSPE-PEG-AC).

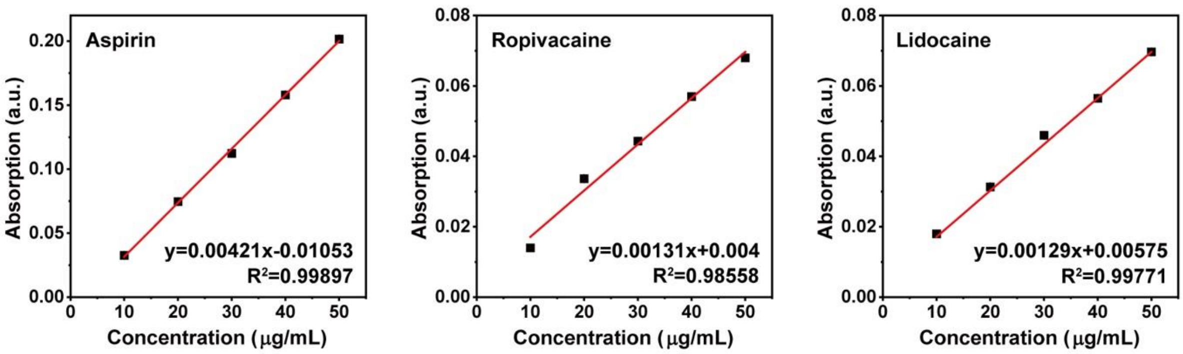

Figure S2. Standard concentration curves for aspirin, ropivacaine, and lidocaine established by UV-Vis spectrophotometry.

Table S1. Reactant dosage of Gel-DNP pre-polymer solution

PEG (Mn = 600) (%wt)	PEGDA (Mn = 700) (%wt)	DNPs solution (%wt)	Darocur 1173 (%wt)
40	19.8	35.4	4.8
60	13.2	23.6	3.2
70	10	17.6	2.4
72	9.25	16.51	2.24
75	8.25	14.75	2
80	6.6	11.8	1.6

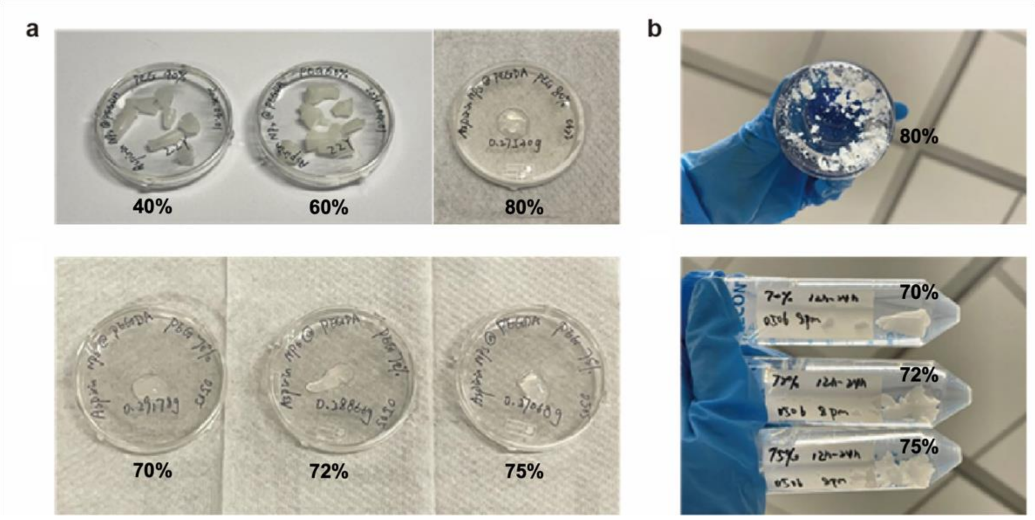

Figure S3. The shape of hydrogels with different PEG ratio before and after soaking. a) Freeze-dried drug-loaded hydrogel with 40%, 60%, 70%, 72%, 75%, 80% PEG; b) The disintegrated hydrogel with 70%-80% PEG after 30 min soaking. The hydrogels with 70% PEG were still in shape, while the hydrogels with 72% PEG were disintegrated in 6h and the hydrogels with 75% and 80% PEG were disintegrated between 1-2h.

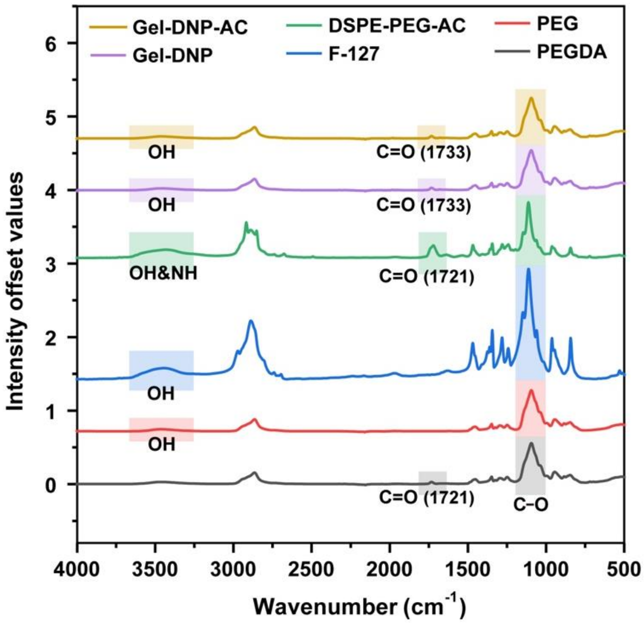

Figure S4. FTIR spectrum of the prepolymer materials (PEG and PEGDA), amphiphilic polymers (F-127 and DSPE-PEG-AC), and both types of hydrogel systems post-polymerization.

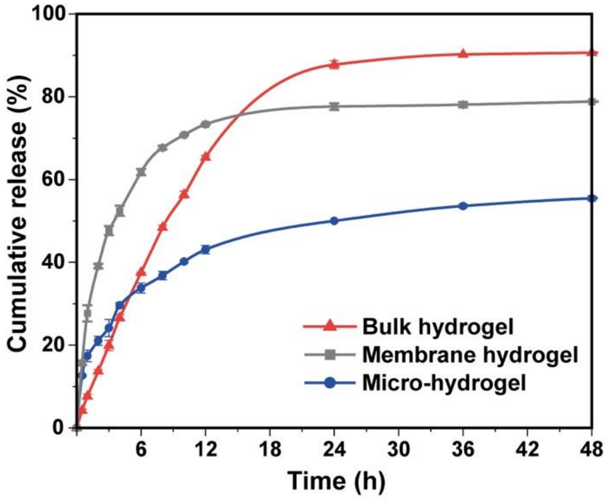

Figure S5. Cumulative release curves of different shapes of hydrogel: bulk, membrane and micro of the PEGDA hydrogels with porogen ratios of 70% crosslinking with DNPs encapsulated by DSPE-PEG-AC.

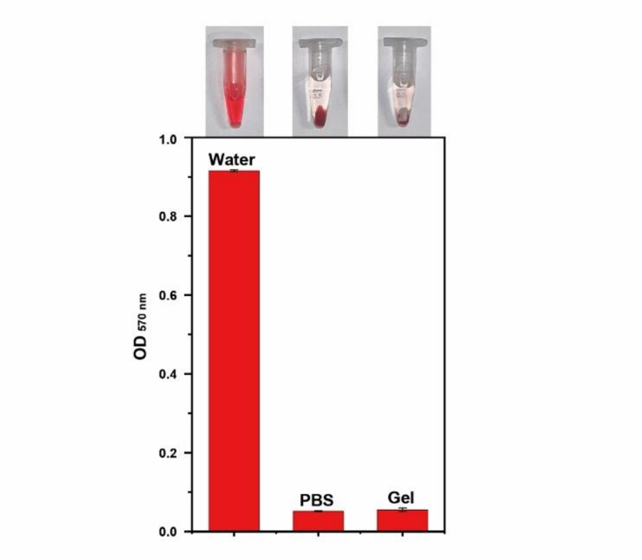

Figure S6. The hemolysis rate of the Gel-DNP-AC.

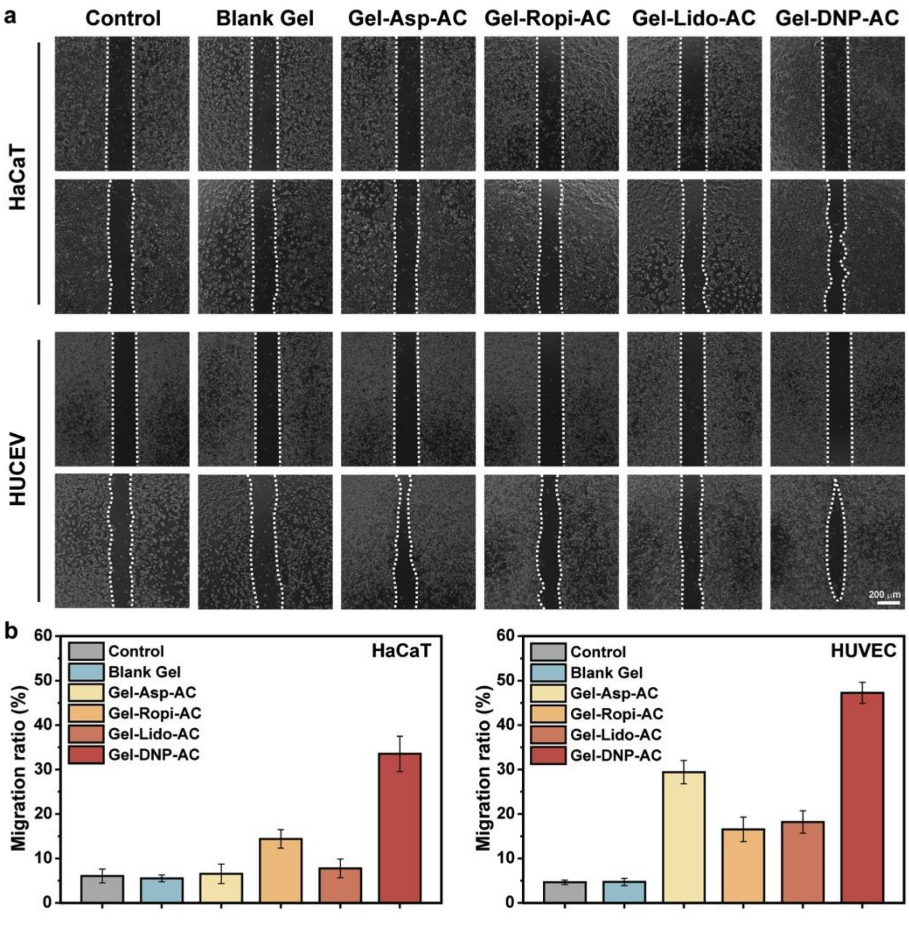

Figure S7. Scratch assays evaluating cell migration. (a) Representative bright-field images of HaCaT and HUVEC scratch assays at 0 h and 24 h post-treatment. Scale bar: 200 µm. (b) Quantitative analysis of cell migration rates.

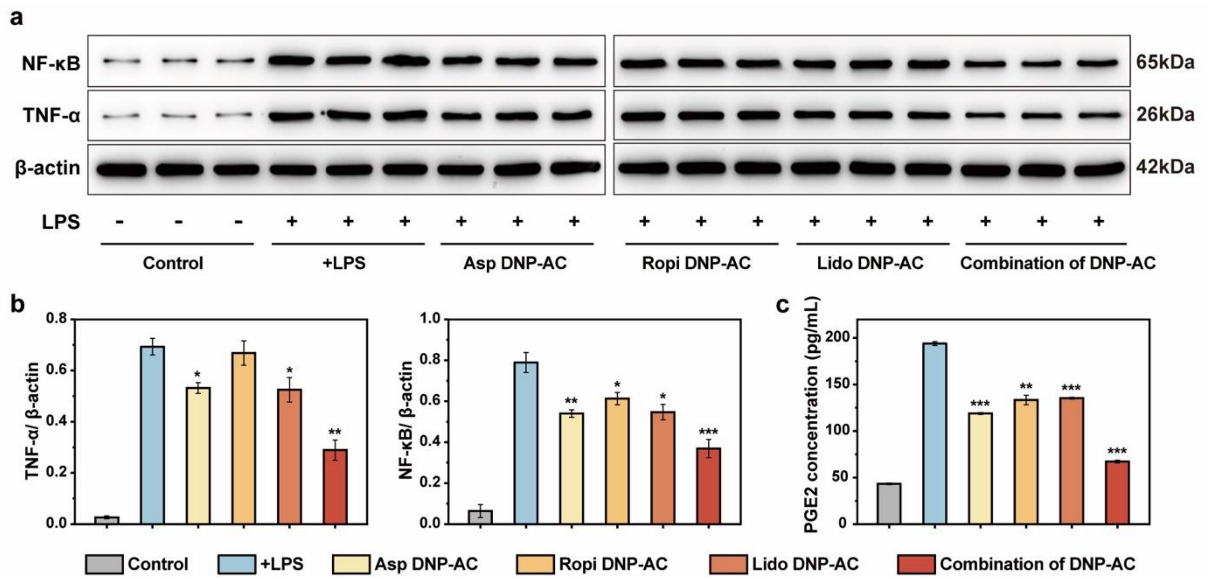

Figure S8. Expression of inflammatory mediators in LPS-stimulated RAW264.7 macrophages. a) Western blot analysis of inflammatory proteins (TNF-α and NF-κB) expression. β-actin was used as a loading control. b) Quantitative analysis of protein expression levels normalized to β-actin (n=3, *p < 0.05, **p < 0.01, ***p < 0.001 vs. LPS group). c) ELISA analysis of pro-inflammatory cytokines (PGE2) secretion in cell culture supernatants (n=3, *p < 0.05, **p < 0.01, ***p < 0.001 vs. LPS group).

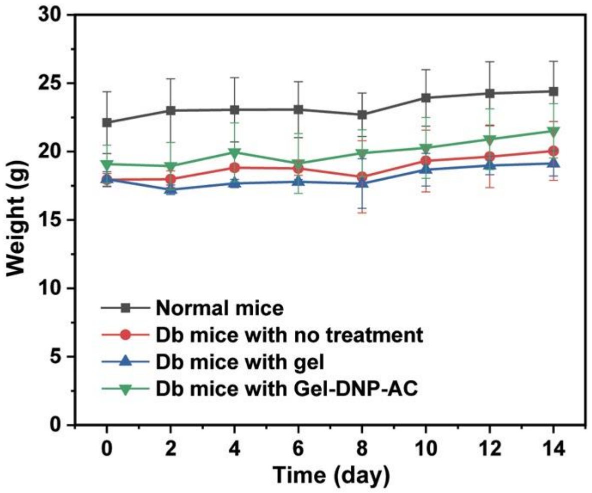

Figure S9. Body weight record of mice during the experiment.

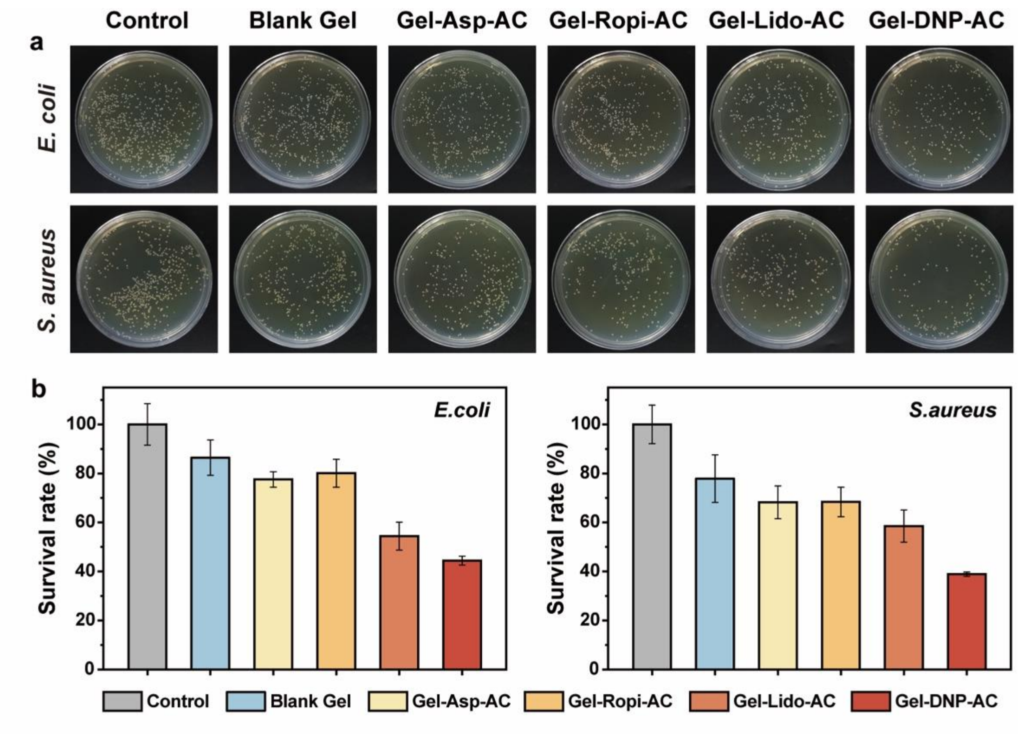

Figure S10. Antibacterial activity of hydrogels against E. coli and S. aureus. (a) Representative images of bacterial colonies after treatment and culture. (b) Quantification of bacterial growth inhibition rates.
